# Molecular and Functional Links between Neurodevelopmental Processes and Treatment-Induced Neuroendocrine Plasticity in Prostate Cancer Progression

**DOI:** 10.3390/cancers13040692

**Published:** 2021-02-09

**Authors:** Roosa Kaarijärvi, Heidi Kaljunen, Kirsi Ketola

**Affiliations:** Institute of Biomedicine, University of Eastern Finland, FI-70210 Kuopio, Finland; roosa.kaarijarvi@uef.fi (R.K.); heidi.kaljunen@uef.fi (H.K.)

**Keywords:** prostate cancer, cancer cell plasticity, neuroendocrine plasticity, treatment-induced neuroendocrine prostate cancer, neuroendocrine differentiation, novel therapies, neurogenesis, axonogenesis, neurobiology of cancer

## Abstract

**Simple Summary:**

Treatment-induced neuroendocrine prostate cancer (t-NEPC) is a subtype of castration-resistant prostate cancer (CRPC) which develops under prolonged androgen deprivation therapy. The mechanisms and pathways underlying the t-NEPC are still poorly understood and there are no effective treatments available. Here, we summarize the literature on the molecules and pathways contributing to neuroendocrine phenotype in prostate cancer in the context of their known cellular neurodevelopmental processes. We also discuss the role of tumor microenvironment in neuroendocrine plasticity, future directions, and therapeutic options under clinical investigation for neuroendocrine prostate cancer.

**Abstract:**

Neuroendocrine plasticity and treatment-induced neuroendocrine phenotypes have recently been proposed as important resistance mechanisms underlying prostate cancer progression. Treatment-induced neuroendocrine prostate cancer (t-NEPC) is highly aggressive subtype of castration-resistant prostate cancer which develops for one fifth of patients under prolonged androgen deprivation. In recent years, understanding of molecular features and phenotypic changes in neuroendocrine plasticity has been grown. However, there are still fundamental questions to be answered in this emerging research field, for example, why and how do the prostate cancer treatment-resistant cells acquire neuron-like phenotype. The advantages of the phenotypic change and the role of tumor microenvironment in controlling cellular plasticity and in the emergence of treatment-resistant aggressive forms of prostate cancer is mostly unknown. Here, we discuss the molecular and functional links between neurodevelopmental processes and treatment-induced neuroendocrine plasticity in prostate cancer progression and treatment resistance. We provide an overview of the emergence of neurite-like cells in neuroendocrine prostate cancer cells and whether the reported t-NEPC pathways and proteins relate to neurodevelopmental processes like neurogenesis and axonogenesis during the development of treatment resistance. We also discuss emerging novel therapeutic targets modulating neuroendocrine plasticity.

## 1. Introduction

An acquired drug resistance occurs in prostate cancer after treatments with next-generation androgen receptor (AR) pathway inhibitors such as abiraterone and enzalutamide (ENZ). Treatment-induced neuroendocrine prostate cancer (t-NEPC) is a variant of prostate cancer which develops during long-term androgen suppression. T-NEPC is castration-resistant and independent of androgen receptor due to a lack of androgen receptor and associated signaling. Generally, in addition to loss of AR signaling, t-NEPC is characterized by neuroendocrine (NE) markers neuron-specific enolase 2 (ENO2, NSE), synaptophysin (SYP) and chromogranin A and B (CHGA and CHGB) [1,2,3]. In addition to t-NEPC, a less common de novo NEPC may also occur [4,5,6]. A recent analysis of 87 NEPC patients revealed that de novo NEPC has worse outcome than t-NEPC but no significant differences on the molecular features were identified between de novo NEPC and t-NEPC [6]. It has been suggested that the de novo NEPC drives from the human prostate luminal epithelial cells [7,8]. However, there are still significant clinical challenges in using NE markers for de novo or t-NEPC diagnosis due to wide variation in the pathologic and diagnostic features of the heterogenous tumors [4].

We and others have reported several novel players underlying and regulating t-NEPC progression. These include the studies on the loss of tumor suppressors TP53, RB1 and PTEN, on the activation of multiple transcription factors including N-Myc (along with AURKA and AURKB activation), ASCL1, SOX2, BRN2, REST, ONECUT2 and CREB, on the role of adrenergic receptors (e.g., ADRB2 and GRK3), on epigenetic modulators and chromatin remodelers (e.g., Polycomb repressive complex 2 proteins EZH2 and SMARCA4), and on the activation of splicing factor SRRM4, or activation of metabolic pathways via PKCλ/ι serine synthesis modulators, among others [1,9,10,11,12,13,14,15,16,17,18,19,20,21,22,23,24,25,26]. Many of these novel NE markers have been identified as overexpressed or activated in NEPC based on DNA or RNA mutation and expression analyses on prostate cancer patient material, also indicating their potential as drug targets [23,27,28,29,30,31,32,33]. However, surprisingly small number of studies have reported their specific cellular functions in prostate cancer cells or utilized specific cellular imaging tools to specifically study the cellular locations and functions of these proteins in NEPC. Additionally, very little is known about the role of these NE markers in cellular communication with the surrounding stroma and the cells in the tumor microenvironment. It is interesting that many of the novel NEPC regulators have previously been studied in the context of neuronal diseases and neurodevelopmental processes. Moreover, several t-NEPC drivers are commonly used as cellular plasticity tools, for example to generate induced pluripotent stem cells (iPSCs, induced by Yamanaka factors SOX2, OCT3/4, KLF4 and C-MYC) or as transcription factors to switch the phenotype of somatic cells into neurons (e.g., ASCL1) [16,34,35]. Still, direct molecular links between neuronal development and regeneration, and neuroendocrine phenotypes in prostate cancer remain largely unclear.

To our knowledge, the first description of prostate cancer-related neurodevelopmental processes axonogenesis and neurogenesis was reported by Ayala et al., in 2008, where the authors identified spatial and temporal associations between increased nerve density and preneoplastic lesions of the human prostate [36]. Although the study focused on the cancer cell-induced axonogenesis and neurogenesis in neurons, it could be hypothesized that also prostate cancer cells utilize axonogenesis and neurogenesis processes for example to connect to surrounding nerve cells. In axonogenesis, axon regrowths from pre-existing, injured neurons through the injury site to re-establish connections [37]. In contrast, neurogenesis refers to the production of new neuronal cells from precursor populations followed by formation of neurites to make connections with host cells [37]. It is still unclear which phenomena is superior in prostate cancer treatment resistance and to what extent but similarities on the phenotypes of axonogenesis and neurogenesis are seen in prostate cancer cells especially in its t-NEPC forms [32,38,39]. This is supported by Grigore et al., who reviewed the interplay between prostate cancer cells and neural structures and hypothesized that in addition to cancer—neuronal crosstalk, a true neural differentiation may also occur in prostate cancer [40]. Recently, a term ‘neurobiology of cancer’ has been proposed to combine these two yet separate research fields together to understand the molecular interactions of cancer cells and neurons, and to silence the cancer—neuronal crosstalk potentially driving the drug resistance. Neurobiology of cancer was first noted in relation to cancer brain tumor resistance and is in the crossroads of oncology and classical neuroscience involving the description of mechanisms how cancer cells build communication networks via neurite-like protrusions to gain neuronal input for growth [41].

Here, we review and discuss potential connections between neuronal regeneration, axonogenesis, neurogenesis and neuroendocrine phenotypic plasticity in prostate cancer. Our goal is to describe the cellular functions of the key regulators and markers of t-NEPC, reveal their known roles in regulating neurodevelopmental processes and potential vulnerabilities which could be utilized to target and prevent the drug resistance and neuroendocrine plasticity in prostate cancer progression to aggressive, treatment resistant disease form.

## 2. Pathways and Proteins Regulating t-NEPC Phenotype and Their Functional Relation to Neurodevelopmental Processes Neurogenesis, Axonogenesis and Synaptogenesis

**Figure 1:**
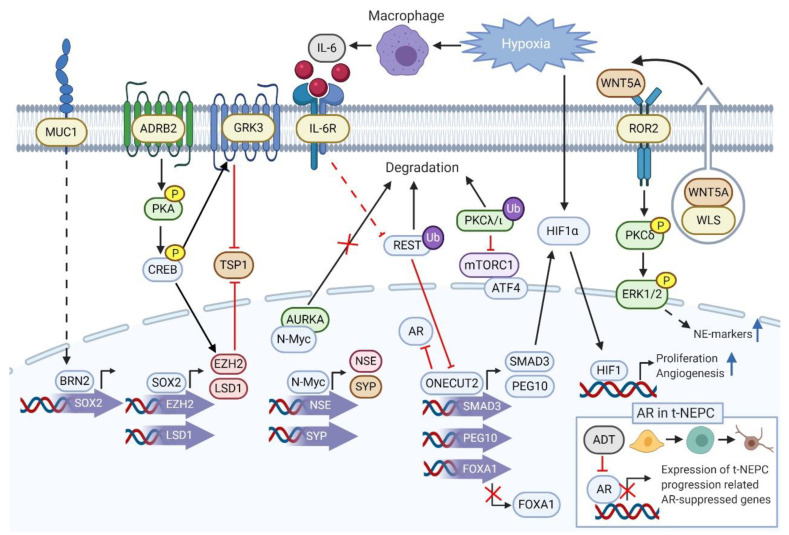
Pathways involved in inducing t-NEPC. BRN2 binds to promoter area of SOX2 increasing its expression. SOX2 in turn promotes expression of epigenetic modulators EZH2 and LSD1 enabling regulation of their NEPC-related target genes. Activation of β-adrenergic receptor, ADRB2 leads to phosphorylation of PKA which phosphorylates and activates CREB. Activated CREB promotes expression of EZH2 and GRK3. GRK3 and EZH2 both have a repressing effect on anti-angiogenic factor TSP1. Increased levels of AURKA are associated with NEPC progression and furthermore AURKA directly interacts with N-Myc preventing its degradation and enhancing expression of N-Myc-regulated neuroendocrine markers NSE and SYP. PKCλ/ι degradation allows mTORC1 complex activation promoting enhanced expression of ATF4-targeted genes involved in t-NEPC progression. Hypoxia also plays a role in prostate cancer progression to its aggressive form. Hypoxia recruits macrophages, which secrete pro-inflammatory cytokine IL-6. IL-6 in turn has on the ability to suppress REST, a negative regulator of genes involved in induction of neuroendocrine differentiation. One of REST targets is ONECUT2, a key driver of NEPC through its ability to promote expression of SMAD3 and PEG10 and to suppress expression of FOXA1. SMAD3 activation allows the modulation of hypoxia related genes through HIF1α to support proliferation and angiogenesis. WLS assists WNT5A ligand secretion and WNT5A binds to ROR2 receptor. ROR2 phosphorylates PKC, which further phosphorylates ERK1/2. Activated ERK1/2 increases expression of NE markers. AR suppresses expression of several proteins involved in t-NEPC progression while this repressive action is diminished upon androgen deprivation therapy (ADT). Transcription markers are marked as light blue, glycoproteins brown, kinases green, receptors yellow, histone methylases red, promoter regions violet, protein complexes light violet, other factors grey, phosphorylation as a yellow p, and ubiquitylation as a dark violet Ub. Figure was created with BioRender.com.

Several emerging pathways and proteins driving t-NEPC have recently been reported in addition to classical NEPC markers SYP, NSE, CHGA and CHGB. An overview of the pathways proposed to be involved in the emergence of t-NEPC is shown in Figure 1.

### 2.1. Lack of Tumor Suppressors TP53, RB1 and PTEN

The roles of tumor suppressors tumor protein P53 (TP53), retinoblastoma protein (RB1) and phosphatase and tensin homolog (PTEN) are well established across a wide spectrum of human malignancies and the key mechanisms conducting their role in suppressing tumorigenesis are well characterized. In relation to neuroendocrine characteristics and t-NEPC, combined double knockout of tumor suppressors PTEN and RB1 or triple knockout of PTEN, RB1 and TP53 in mouse models reveal phenotypic plasticity and prostate cancer resistance to therapeutics, exhibit stem-like and neuroendocrine differentiation features and loss of AR activity [10,13,42]. In addition, AR and the E2F-RB1 pathway dynamically regulate paternally expressed 10 (PEG10) isoforms during the NEPC development [11]. PEG10 promotes cell-cycle progression in the absence of TP53 and regulates Snail expression via TGF-β signaling to promote invasion [43]. Though the combined loss of TP53 and RB1 is not sufficient to uniformly induce neuroendocrine phenotypes in prostate carcinoma, it is nevertheless interesting that disruption of RB1 expression in nerve cells in vitro has been shown to enhance the growth of nerve cells and adult nerve regeneration [9,44]. However, the central axon pathways affected by RB1 knockdown are unknown. Moreover, the loss of TP53 drives neuron reprogramming in mouse models of head and neck cancers where the loss of TP53 alters the neural microenvironment throughout tumor evolution [45]. These findings on the roles of RB1 in neuronal dendrite plasticity and axon regeneration and TP53 in neuron reprogramming raise questions whether similar phenotype-switching mechanisms orchestrated by RB1 or TP53 also trigger neuroendocrine phenotype switch in prostate cancer, and whether-and how-the neuron-like phenotype provides advantage for survival and protection from current anti-cancer therapies.

### 2.2. Transcription Factors Driving Treatment Resistance and NEPC Phenotype

#### 2.2.1. BRN2 and SOX2

BRN2 (POU3F2) belongs to the class III POU (Pit-Oct-Unc)-domain containing octamer-binding reprogramming transcription factors which regulate the expression of genes required for cell lineage determination and are also key factors in neurogenesis [46,47,48]. BRN2 together with other POU3F transcription factors has been shown to promote neurogenesis by regulation of Notch-signaling resulting in upregulation of proneural genes [46,47]. Furthermore, to emphasize the importance of BRN2 as lineage-determining factor, combination of BRN2, basic Helix-Loop-Helix (bHLH) transcription factor Achaete-scute homolog 1 (ASCL1) and Myelin transcription factor 1 like (Myt1l) enabled not only the conversion of mouse fibroblasts into functional induce neuronal (iN) cells but also generation of iN-cells from human fibroblast cells [49]. Interestingly, BRN2 alone is sufficient to induce reprogramming of astrocytes into neural progenitor cells or further into neurons [50]. BRN2 has also been identified as a key regulator in cancer progression, in particular in driving neuroendocrine differentiation [17,51]. We recently reported BRN2 as a master regulator driving t-NEPC and ENZ resistance by directly regulating NEPC marker sex determining region Y-box 2, SOX2 [17]. BRN2 itself is under direct suppressive regulation of AR [17]. Interestingly, a recent study revealed that the induction of neuroendocrine differentiation by BRN2 involves release of BRN2 and another POU3F transcription factor, BRN4 in prostate cancer extracellular vesicles [52]. SOX2 is a transcription factor that is important for maintenance and function of neural and embryonic stem cells (NSCs and ESCs) through its ability to control cellular pluripotency and self-renewal [53,54,55]. The pluripotency in ESCs is controlled by a transcriptional regulatory circuitry, in which SOX2 has a vital part in addition to transcription factors OCT4 and NANOG [55]. Additionally, in ESCs, SOX2 promoter shares the same promoter binding sites with OCT4, whereas in NSCs SOX2 co-occupies promoter sites with BRN2, providing insights into the regulation of these two distinct stem cell populations by SOX2 [18]. Interestingly, SOX2, BRN2 and FOXG1 can also transdifferentiate fibroblasts to neural precursor cells [56]. Moreover, a recent discovery reveals that three neuronal transcription factors ASCL1, BRN2, and Myt1l (BAM factors) are sufficient to convert mesodermal fibroblasts or endodermal hepatocytes into fully functional neuronal cells [57].

The role of SOX2 in prostate cancer is well-established and it has been shown that SOX2 is one of the key drivers of lineage plasticity promoting drug resistance associated with aggressive forms of prostate cancer [10]. The expression of SOX2 is under regulation of androgen receptor, AR: AR binds to enhancer element in promoter of SOX2 leading to suppression of SOX2 expression [17]. Recently, a novel mechanism for SOX2 in NEPC progression was presented involving lysine-specific demethylase 1 (LSD1) -mediated global epigenetic modulation. This would open a window of opportunity for developing anti-cancer drugs targeting LSD1 and through it, cancer cells with high SOX2 [58].

#### 2.2.2. ASCL1

ASCL1 (also known as mASH1 or hASH1) is an activator-type pro-neural bHLH transcription factor of the neurogenin family that has been reported as overexpressed in neuroendocrine tumors of several malignancies, including in medullary thyroid cancer, small cell lung cancer and prostate cancer [59,60,61,62]. Although the role of ASCL1 in neuroendocrine differentiation is not fully understood, it is known to drive the differentiation of neural progenitor cells and their commitment to neuronal lineages [16]. As a master regulator of neurogenesis, ASCL1 targets include a variety of transcriptional regulators but also cytoskeleton-related proteins with essential functions in neuronal differentiation program [63,64]. Additionally, ASCL1 is also involved in maintaining cell proliferation of neural progenitors through its ability to directly activate genes which promote cell cycle progression [65]. ASCL1 like other proneural genes can inhibit its own expression in the adjacent progenitor cells through activation of Notch signaling pathway, and concomitant expression of Hes1 and Hes5 proteins, which function as repressors of transcriptional activators, such as ASCL1, thus inhibiting neuronal differentiation [66]. Recently, ASCL1 was reported to directly reprogram and induce a phenotype switch of somatic stem cells to functional neurons either alone or in combination with SOX2 and NEUROG2 suggesting that similar reprogramming may occur also in prostate neuroendocrine lineage reprogramming [34,67]. Interestingly, ASCL1 and NEUROD1 also regulate different genes that commonly contribute to neuronal function in pulmonary neuroendocrine tumors, a phenomenon which may also occur in prostate cancer [15].

#### 2.2.3. ONECUT2

ONECUT2 gene (Oc2 in mammals) encodes a transcription factor that consists of a bipartite DNA-binding domain with a single cut domain and a homeodomain (HD) [68]. ONECUT2, like other mammalian Onecut protein family members functions as a transcriptional activator controlling cell differentiation in liver and pancreas and has also been indicated to participate in regulation of the early steps of motor neuron differentiation [20]. Additionally, ONECUT2 is necessary for the development of the mouse retina, more specifically in the formation of normal number of horizontal cells [69]. The importance of ONECUT2 in prostate cancer progression has been recently recognized and its role in NEPC has been further elucidated. To this end, ONECUT2 was shown to act as suppressor of AR activity and to directly activate PEG10 thus supporting the central role of ONECUT2 in the transition from adenocarcinoma to NEPC [70]. Gene expression profiling in prostate cancer cell lines modeling aggressive prostate cancer revealed enrichment of genes related to cell motion, neuronal differentiation, and mesenchymal cell differentiation among the ONECUT2-induced genes [70]. ONECUT2 was also found to bind the FOXA1 promoter and repress both mRNA and protein expression of FOXA1, an inhibitor of neuroendocrine differentiation [70,71]. The expression of ONECUT2 on the other hand is regulated by REST through direct repression upon binding of REST to ONECUT2 promoter [70]. In a study by Guo et al. ONECUT2 was identified as key driver of NEPC based on a pan-NET analysis that incorporated two adeno-CRPC and NEPC datasets but also data from malignancies of lung and nervous system [24]. Additionally, it was shown that ONECUT2 induces pathways related to angiogenesis and hypoxia, both of which are linked to NEPC progression. In particular, ONECUT2 was found to activate SMAD3, a regulator of hypoxia-signaling, and consequently modulate the binding of hypoxic response factor HIF1α to chromatin, allowing the regulation of hypoxia-related genes to enhance cell proliferation and angiogenesis [24]. To support the synergy between ONECUT2 overexpression and hypoxia in inducing NEPC, combining these two resulted in reduced AR signaling and enhancement of NE-like cell morphology, which was also observed by overexpression of ONECUT2 alone.

Taken together, the experimental data available demonstrate that ONECUT2 is a key player in prostate cancer progression into its most aggressive form, NEPC. Targeting ONECUT2 itself might be problematic due to its expression also in normal tissues, such as brain, liver, and pancreas but promising results have been observed when targeting ONECUT2-dependent tumor hypoxia [24].

#### 2.2.4. REST

Repressor element 1 silencing transcription factor (REST, also known as neuron-restrictive silencing factor or NRSF) is a transcription factor that functions as repressor of a large set of neuronal genes in non-neuronal cells [72,73]. The gene regulatory role of REST is also vital during neurogenesis where REST regulates the transition to progenitor cell and further to mature neuron. This process is associated with modulation of REST protein levels to allow adjustment of neuronal gene activation, culminating to the loss of REST during the final stages of neuronal differentiation [74].

The cellular levels of REST are regulated by ubiquitin-mediated proteolysis through the action of E3 ubiquitin ligase β-TRCP, which targets REST for degradation and is known itself to be upregulated during oncogenic transformations [75]. In prostate cancer, loss of REST is associated with the emergence of the most aggressive form of the disease, NEPC, featuring its known characteristics, notably the loss of AR signaling, and induction of genes related to neuroendocrine differentiation, such as CHGA, a target gene of REST [74]. REST expression negatively correlates with prostate cancer recurrence and mediates AR associated gene repression [19]. The role of REST in NEPC has been further supported through its ability to suppress interleukin-6 induced neuroendocrine differentiation in prostate cancer cells [76]. Paracrine interleukin-6 (IL-6) is involved in the induction of neuroendocrine differentiation and mediates the associated features, such as acquisition of a neurite-like phenotype and growth arrest in prostate cancer cells.

REST has also been linked to the PI3K/AKT pathway which promotes tumor progression and resistance to treatment when aberrantly active in many human cancers. Combined inhibition of both AKT and AR in prostate cancer cells results in reduction of REST expression and upregulation of t-NEPC specific REST target genes, thus indicating that induction of neuroendocrine differentiation of prostate cancer upon AKT pathway inhibition is mediated by REST protein degradation [77].

#### 2.2.5. FOXM1

Forkhead box protein M1 (FOXM1) is a transcription factor with multiple functions in several cancer and stem cells [78,79]. In addition to its role in regulating cell proliferation, it acts as a major regulator of pluripotency factors SOX2, OCT4 and NANOG as well as Aurora kinases A and B (AURKA and AURKB) which are common markers of neuroendocrine prostate cancer [2,33,80,81]. FOXM1 is also able to change the cancer epigenome in breast cancer [82]. We recently described FOXM1 as a master regulator activated in highly aggressive prostate cancer patient subtype displaying neuroendocrine signature, and monensin as a novel FOXM1 inhibitor [83]. The FOXM1 inhibition reduced stemness and growth of enzalutamide resistant prostate cancer tumors with stem and neuroendocrine-like phenotype in vivo [83]. In relation to neuronal developmental processes, FOXM1 has been reported to be required for both proliferation and differentiation of neuronal precursors in early *Xenopus* embryos, it regulates proliferation during brain development and potentially regulates neural stem cell proliferation and differentiation [84,85,86]. Although direct links to neurodevelopmental processes and its specific role in neuroendocrine transdifferentiation have not been described, FOXM1 regulates several known NEPC molecules including SOX2, AURKA and AURKB indicating a potential role also in regulation of t-NEPC.

#### 2.2.6. N-Myc

N-Myc proto-oncogene protein (N-Myc) is a regulator of neurogenesis in early embryonic developmental stages and becomes downregulated as neurons mature. N-Myc is mainly present in progenitor cells and it contributes to maintenance of pluripotency [87]. Upregulation and amplification of N-Myc is detected in 40% of NEPC tumors whereas it is present in only 5% of prostate adenocarcinoma tumors suggesting that N-Myc contributes to the development of NEPC [33]. RNA-sequencing analysis from mouse overexpressing human N-Myc showed high enrichment of genes contributing to epithelial-mesenchymal transition (EMT) while in cell line-based RNA-analyses, downregulation of androgen signaling was detected [25]. This suggests that N-Myc is an important driver of cellular plasticity in prostate cancer upon the emergence of androgen-independence. More detailed investigation of N-Myc target genes using chromatin immunoprecipitation revealed that N-myc binds to promoter regions of NSE and SYP as well as AR [25]. Additionally, it was noticed that N-Myc physically interacts with Aurora kinase A (AURKA) and improves AURKAs stability [33]. Moreover, EZH2 expression is under regulation of N-Myc, and EZH2 is a critical epigenetic modulator of the development of neuroendocrine prostate cancer [33]. N-Myc has been a target for drug development for a long time due to its relevance in many highly aggressive cancers. However, due to structural challenges of MYC proteins, they have been considered as poor drug targets. Therefore, alternative approaches have been taken, and for example CD532, a dual-inhibitor of N-Myc and AURKA, has been also studied as a suitable drug for neuroendocrine prostate cancer [80,81].

### 2.3. Mitotic Spindle Proteins Aurora Kinases A and B

Prostate cancer, like many other malignancies, is featured by chromosomal instability that has been linked to defects in mitotic regulations and thus induced aneuploidy in cancer cells. Serine/threonine protein kinases of the Aurora family, namely Aurora kinases A and B (AURKA and AURKB) are important regulators of mitotic events functioning in mitotic spindle formation (AURKA) and chromosome segregation (both AURKA and AURKB) [88]. Increased expression levels of both AURKA and AURKB have been observed in prostate cancer promoting cell proliferation and correlating with higher malignancy [89,90]. In prostate cancer AURKA has been shown to block the degradation of the transcription factor N-Myc, and the cooperative function of these two drives the progression prostate cancer [80,91]. AURKA has also been shown to promote survival of prostate cancer cells by suppressing autophagy and furthermore the autophagy-induced apoptosis through inhibition of Akt phosphorylation [92].

AURKA has also been implicated in non-mitotic functions, in addition to its more highlighted role in regulation of mitosis related events. To this end, an atypical protein kinase C (aPKC)-AURKA-NDEL1 pathways was shown to play a crucial role in regulation of microtubule organization during neurite extension [93]. This discovery was supported by the observed decrease in neurite extensions of bipolar cortical neurons and upon the depletion of AURKA (or aPKC) [93]. Additionally, the microtubule dynamics were negatively affected by AURKA depletion [93]. The role of AURKA as regulator of microtubule organization via aPKC-AURKA-NDEL1 pathway was additionally shown to be essential for neuronal migration revealing an interplay between CDK5 and AURKA [94].

Interestingly, also AURKB has been associated with neuronal functions through the discovery of a previously unrecognized role for AURKB as a regulator of mitochondrial trafficking in neurons [95]. This finding was supported by the observation that AURKB knockdown promoted mitochondrial axonal transport in both rat hippocampal neurons and in induced pluripotent stem cell (iPSC)-derived human cortical neurons [95]. As cancer-associated kinases, both AURKA and AURKB have been extensively studied in search for new potential small molecular inhibitors that could be used in anti-cancer therapy (reviewed by Borisa and Bhatt) [96]. Targeting only a selected kinase has not been an effective strategy so far but promising results have been obtained from combinatorial treatment with kinase inhibitor and chemotherapeutic drug.

### 2.4. Epigenetic Modulators and Chromatin Remodelling Complex Members and Their Regulators

#### 2.4.1. EZH2, CREB and GRK3

Enhancer of Zeste Homolog 2 (EZH2) is a critical player in the early steps of neuroendocrine differentiation in prostate cancer [23]. Its expression and function are regulated by several main transcription factors involved in transdifferentiation, such as SOX2 [97]. EZH2 is part of polycomb repressive complex 2 (PRC2) and as a methyltransferase, it tri-methylates H3K27 inducing a change from self-renewal state to differentiative state in progenitor cells [97]. As EZH2 has been shown to be a critical modulator of initiation of transdifferentiation, it is an intriguing target in drug development. EZH2 inhibitor, GSK343, was shown to suppress stem-like state of cancer cells in glioma cells and to reverse EMT [98]. A study of genetic targets of EZH2 revealed that CREB/EZH2/TSP1 pathway is important for the t-NEPC progression as EZH2 suppressed anti-angiogenic factor thrombospondin-1 (TSP1) [21]. Interestingly, EZH2 regulates the balance between self-renewal and differentiation in the cerebral cortex indicating that EZH2 also has a role in neuronal development [99]. Currently, three EZH2 inhibitors, GSK2816126, tazemetostat (EPZ 6438) and CPI 1205 are studied in clinical trials for NEPC (clinicaltrials.gov (accessed on 18 December 2020)).

CREB, cAMP response element-binding protein, is a transcription factor originally recognized as a regulator of neurodevelopment, neuronal plasticity and neuroprotector [100]. The activity of CREB is controlled through phosphorylation and phosphorylated CREB binds to cAMP response elements to regulate the transcription of its target genes [100]. Several kinases are capable of CREB phosphorylation and for example the induction of β-adrenergic (ADRB)/PKA/CREB and -pathway has recently been studied more closely in the context of NEPC [21,100]. Zhang et al., report that ADT activates CREB, and induced CREB phosphorylation is required for the NEPC phenotype as inhibition of CREB activation through β-adrenergic and PKA inhibitors leads to downregulation of NE markers [21]. The induction of neuroendocrine differentiation by CREB involves enhancement of EZH2-mediated epigenetic repression of its target genes, including TSP1, an inhibitor of angiogenesis and tumor growth. [21,101]. Another mechanism of action for CREB is targeting G protein coupled receptor kinase 3 (GRK3), which is also known as β-adrenergic receptor kinase [21,22]. Moreover, ADT-induced CREB-activation promotes the expression of GRK3 which in turn induces NE phenotype [22]. A direct inhibitor for CREB, 666-15, is under preclinical investigation [102]. If not targeting CREB directly, inhibitors for β-adrenergic receptors are also a potential way to decrease CREB activity. Currently, targeting β-adrenergic receptors as a treatment for NEPC is under investigation.

#### 2.4.2. SMARCA4

SMARCA4 (also known as Brg1) is one of the two ATPase units of the mammalian SWI/SNF chromatin remodeling complex, which has a central role in transcriptional regulation through controlling chromatin accessibility acting in regulation of lineage specificity and cell fate determination [103]. SMARCA4 itself is an essential component in neurogenesis as it has been shown that in addition to binding to the bHLH transcription factors neurogenin1 (Ngnr1) and NeuroD, the ability of these transcription factors to drive neuronal differentiation is diminished upon the loss of SMARCA4 (Brg1) function [104]. SMARCA4 has also been presented as potential regulator of the switch from neurogenesis to gliogenesis through its action as a repressor of neuronal differentiation in neural stem cells (NSCs) [105]. Many SWI/SNF subunits have been shown carry inactivating mutations in different cancers thus supporting the role of this chromatin remodeling complex as regulator of tumorigenesis [106]. Recently, based on a genome-wide study by Cyrta et al., on the role of SWI/SNF in NEPC, a potential tumor-promoting function of this chromatin remodeling complex was shown [107]. In particular, increased expression of the SMARCA4 (Brg1) subunit was associated with the aggressive neuroendocrine phenotype of prostate cancer, marked by increased NE marker expression and shorter overall survival [107]. Furthermore, SMARCA4 was shown to interact with several factors specific to neural differentiation, including the transcription factor NKX2.1 (also known as TTF-1) and the growth factor VGF, indicating the involvement of SMARCA4 and thus the SWI/SNF complex in NEPC-related neurogenesis [107].

Due to the recognized contribution of SWI/SNF complex and its components in cancer, targeting this complex or pathways/molecular units associated with it has become a topic of research, as reviewed recently by Mittal and Roberts [108]. The potential of SMARCA4 and its mutually exclusive paralog SMARCA2 as therapeutic target has been already investigated in the case of lung cancer using an allosteric SMARCA4/SMARCA2 inhibitor [109]. The results of this study were promising showing downregulation of SMARCA2-dependent gene expression and antiproliferative activity in lung-tumor-xenograft. As another potential therapeutic option relating to SMARCA4 overexpression in cancer, comes from the use of protein degraders, namely proteolysis targeting chimeras (PROTACs) designed to target proteins to ubiquitin proteosome system for degradation [110]. The PROTAC-induced knockdown of SMARCA2/4 was shown to have a noticeable antiproliferative and apoptosis-inducing effect, supporting its potential as anticancer therapy option.

### 2.5. Receptors

#### 2.5.1. Steroid Receptors

Very central role in prostate cancer treatment-resistance is the steroid and nuclear receptor, androgen receptor, AR, itself: AR activity and its target genes are either lost or blocked in t-NEPC [1,23]. We and others have suggested that AR has a suppressive role in regulating NEPC phenotype and neuron-like targets genes and consequently targeting AR allows an upregulation and/or activated AR suppressed genes and AR suppressed transcriptional complexes [111]. Moreover, there are evidences shown that AR cooperates with other steroid receptors like glucocorticoid receptor (GR) [112,113,114]. Future studies are needed to understand the cooperation and co-suppression mechanisms of AR, GR and other steroid receptors at chromatin level and whether these co-suppression mechanisms contribute to t-NEPC progression. It is also unclear whether AR suppression has consequences on neuron-like phenotype similarly as is seen in neurons where AR has a central role in regulating neuronal development, regeneration and plasticity [115,116]. There is a lack of understanding of the similarities and differences between AR regulated cellular phenotypic changes in neurons and prostate cancer cells. In neurons, testosterone and activated AR signaling is linked to induced neurogenesis, although in Alzheimer’s disease, controversial results have been reported [115,116,117]. As t-NEPC phenotypic plasticity is evidenced by suppressed AR activity, and morphologically AR suppressed NEPC-like cells adapt a neuron-like phenotype [22], it is interesting that in addition to classical sites in the cell nucleus, AR protein is located in axons and dendrites of the amygdala and cerebral cortex in brain [118]. Thus, AR may play roles in rapid behavioral effects of androgens in these non-classical AR sites in axons and dendrites [118].

Future studies are needed to understand whether AR has similar effects in neuron-like t-NEPC phenotype as in neurons, which molecular mechanisms contribute the phenotypic plasticity in t-NEPC cells and whether these phenomena have any role in maintaining treatment resistance in prostate cancer. For example, it would be interesting to find out whether AR has a similar specific role in cytoplasm as it has in neurons—especially in axon and dendrite-like structures of neuron-like t-NEPC cells where AR translocation into nucleus is blocked by ADT and potentially still expressed AR protein remains in the cytoplasm. Interestingly, in addition to canonical AR signaling in the nucleus, non-genomic AR signaling occurs in the cytoplasm [119]. In penile carcinomas, non-genomic AR signaling has been suggested to play a role and to correlate with poor prognosis and relate to shorter overall survival [120]. It is unknown whether the cytoplasmic AR attracts signals from the surrounding stromal cells or sympathetic nerves located in the prostate cancer tumor microenvironment. The cytoplasmic AR binds to several proteins and activates several signaling molecules including SRC, RAS, MAPK, AKT, EGFR, and PI3K among others in prostate cancer [120,121,122]. As AR inhibitor ENZ leads to accumulation of high levels of cellular cytosolic AR, it is interesting to found out whether the high levels of cytosolic AR have any specific role in ENZ-induced NEPC transdifferentiation [123]. Currently, there are no FDA approved antagonists available to target cytosolic AR [122].

Although it is tempting to hypothesize that the cellular switch into neuroendocrine, neuron-like phenotype is driven by AR, it is necessary to remember that at the final stage of differentiated NEPC, the prostate cancer cells have been reported to completely lack AR expression [124]. Thus, the specific timing when and how AR is completely lost in the transdifferentiation process and whether it has any mechanistic role in the phenotype switch needs further investigation.

#### 2.5.2. Adrenergic Receptors

Neuroendocrine transdifferentiation is also induced by β2-adrenergic receptor (ADRB2) signaling: tumors with low pre-treatment ADRB2 levels are able to resist androgen-targeted therapy through improved maintenance of androgen levels [125]. As low-ADRB2 tumors are unable to undergo ADT-induced neuroendocrine differentiation, low-ADRB2 tumors represent a model for androgen-driven CRPC adenocarcinoma [125]. Moreover, as ADRB2 is essential for ADT-induced neuroendocrine differentiation, the results suggest that high-ADRB2 tumors are more likely to develop aggressive, androgen-indifferent prostate cancers like t-NEPC [125]. ADRB2 is a member of ADRB family proteins with 7-transmembrane G-protein coupled receptors which makes out a part of the sympathetic nervous system [126,127]. It is very important regulator of rapid stress response and energy expenditure with its endogenous ligands, neuropeptides epinephrine and norepinephrine [126,127]. Interestingly, norepinephrine is primarily produced in axon terminals and is with epinephrine stored in secretory vesicles with NEPC marker CHGA [128]. Norepinephrine is secreted by the sympathetic nerves which are located in the prostate cancer tumor microenvironment and stimulate ADRB2 on cancer cells [129]. Prostate cancer cells on the other hand secrete neurotrophic growth factors which can stimulate axonogenesis [130]. Though it is now known that ADRB2 stimulation induces t-NEPC, the underlying mechanism how the morphological changes are regulated and how neurite outgrowth in prostate cancer cells is controlled requires further studies. Since epinephrine and norepinephrine potentiate cancer growth and resistance to treatment in many types of cancers, it can be hypothesized that neuroendocrine plasticity may lead to increased catecholamine synthesis in prostate cancer tissue. Chronically elevated catecholamines induce neuroendocrine differentiation of prostate epithelial cells and it has been suggested that immune cells and neurodifferentiated prostate cells could secrete epinephrine and/or norepinephrine and thus trigger the sustained ADRB2 signaling in prostate tumors [126,127]. Although prostate cancer progression has been shown to be dependent on the development of autonomic nerves into the tumor microenvironment, the role of tumor microenvironment and how surrounding nervous system contribute to specifically t-NEPC progression needs further investigation [131].

#### 2.5.3. ROR2

Several signaling pathways have been studied for decades in the context of their role in inducing or bypassing androgen receptor signaling in castration and/or treatment resistant prostate cancer. Although many signaling pathways are under investigation in NEPC, surprisingly little is known about their specific roles in the development of t-NEPC phenotypes. Receptor tyrosine kinase like orphan receptor 2 (ROR2) is one of the very recently identified receptors overexpressed and activated in t-NEPC [132]. ROR2 is a transmembrane protein and a noncanonical Wnt receptor and a favored receptor for Wnt5A in noncanonical Wnt5A signaling leading to morphogenetic movements during embryogenesis [133]. Interestingly, the absence of Ror signaling leads to decreased branching of sympathetic neuron axons [133]. ROR2 also plays a role in Wnt5a-induced depolarization of hippocampal neurons and increases neuronal excitability [134]. In the context of t-NEPC, Bland et al., report that both ROR2 and Wnt5a ligand transporter Wntless are overexpressed in enzalutamide-resistant NEPC-like cells and Wntless is directly suppressed by AR leading to Ror signaling activation via ROR2/PKCδ/ERK signaling pathway to support NEPC-like cell proliferation [132]. The authors also propose Wnt pathway inhibition as a potential targeted therapy for NEPC [132].

### 2.6. RNA Splicing Factors; the RNA Splicing Factor Serine/Arginine Repetitive Matrix 4, SRRM4

Alternative splicing is also detected both in prostate cancer neuroendocrine differentiation and neurogenesis. In neurogenesis, alternative splicing occurs in brain tissues at high frequency and it contributes to all steps of neurodevelopment processes including cell-fate decisions, neuronal migration, axon guidance and synaptogenesis [135]. The neuron-specific splicing regulators need to be timely expressed in neuronal development processes [135]. One of the RNA splicing factor, RNA splicing factor serine/arginine repetitive matrix 4, SRRM4, was recently identified as a strong stimulator of adenocarcinoma cells to express NEPC biomarkers under androgen receptor pathway inhibition. The SRRM4-targeted genes REST and PHF21A were identified in NEPC patient samples which suggests that SRRM4 is functionally active in NEPC. Interestingly, in neurons, SRRM4 regulates neural-specific exon networks which are required for embryonic stem cells to transdifferentiate into neural cells [136,137]. Although the detailed mechanisms how SRRM4 is induced in neuroendocrine transdifferentiation to drive NEPC under androgen deprivation, the results indicate that in addition to playing important roles in nervous system developmental processes including regulation of morphogenesis and synaptic plasticity and the formation of complex neuronal networks, alternative splicing may play an important role also in maintaining neuron-like phenotype in aggressive form of prostate cancer.

### 2.7. PKCλ/ι and Serine Synthesis Modulators

The role of cellular metabolic pathways and metabolic reprogramming in relation to neuroendocrine plasticity and neuron-like phenotype are yet to be clarified. Interestingly, Reina-Campos et al., reported recently that PKCλ/ι deficiency-increased serine and one-carbon pathway metabolism promotes the neuroendocrine prostate cancer development [26]. The authors report that PKCλ/ι loss promotes a metabolic reprogramming in t-NEPC: the PKCλ/ι C is downregulated in de novo and during therapy-induced NEPC resulting in the upregulation of serine biosynthesis through an mTORC1/ATF4-driven pathway [26]. The authors conclude that this metabolic reprogramming supports cell proliferation and increases intracellular S-adenosyl methionine levels to feed epigenetic changes that favor the development of NEPC characteristics [26].

As an interesting detail, the authors reported that blocking of retrograde transport, that moves physiological materials back to the cell body from the periphery with dynein inhibitor Ciliobrevin D, reduced the induced mTORC1 activity in PKCλ/ι deficient cells which maintain perinuclear aggregation of lysosomes and display NEPC phenotype [26]. These results lead to a hypothesis that regulation of lysosomal distribution and dynamics in the cells as well as control of cellular transport systems, which are also very important neuronal processes maintaining neuron polarity and synaptic plasticity, are also important in maintaining the NEPC phenotype. However, more studies are needed to confirm whether the metabolic reprogramming drives neuron-like phenotypic plasticity in NEPC or if these are separate processes observed during NEPC progression.

### 2.8. Cell Surface Membrane-Anchored Proteins

#### 2.8.1. MUC1

Mucin 1 (MUC1) is a membrane tethered glycoprotein that consists of N-terminal subunit (MUC1-N) and C-terminal transmembrane subunit (MUC1-C) and is normally expressed in the glandular or luminal epithelial cells of the mammary gland, pancreas, prostate and lungs, among others [138]. Aberrant overexpression of MUC1 has been linked to several human carcinomas contributing to the known characteristics of cancer cells, including EMT, stemness, resistance to anti-cancer treatments, epigenetic programming, and immune evasion [14,139,140,141]. Recently, it was shown that upregulation of MUC1-C in androgen-dependent prostate cancer cells leads to suppression of AR axis signaling and induces the neural BRN2 transcription factor [142,143]. Additionally, MUC1-C was also found to suppress the TP53 pathway, to induce expression of pluripotency factors, such as SOX2 and MYC, and to drive stemness [142]. These, together with earlier findings of MUC1-C role in activation of EMT related signaling pathways and promoting EMT by disrupting cell polarity and cell-cell interactions, provide evidence for MUC1-C as key player in NEPC progression. To support the clinical potential of MUC1-C there is experimental data available showing that targeting MUC1-C with GO-203 inhibitor to block MUC1-C homodimerization and nuclear localization leads to inhibition of BRN2 signaling, the NE phenotype, self-renewal capacity and tumorigenicity both in vitro and in prostate cancer tumor xenograft models [142,143]. Interestingly, MUC1 is also an important signaling protein in neural tracking and plays a role in perineural invasion of cancer cells which supports future investigations on the tumor—neuron interactions in t-NEPC progression [144].

#### 2.8.2. CD44

Cell surface bound proteins are important players in cell signaling and cell-cell communications within the tumor microenvironment. Another heavily studied membrane protein is CD44 which is transmembrane glycoprotein, also known as homing receptor, that mediates adhesive cell–cell and cell–matrix interactions [145]. Although CD44 has widely utilized as a cancer stem cell marker in several cancers including prostate cancer, the functional role of CD44 beyond cancer stem cell marker has not been heavily studied in NEPC except a recent study reporting that CD44 plays a role in glucose metabolism in NEPC [146,147]. Moreover, our recent data also reveals that CD44 is a functional molecule on the surface of extracellular vesicles affecting their physical properties, homing, binding, and signaling to targets, proposing that CD44 plays variety roles in cellular communication and potential neuron-like processes in prostate cancer progression [148]. As CD44 has a role in molecular interactions, signaling and functions in the nervous system, novel functional roles in relation to its role in prostate cancer progression and neuron-like phenotype may be discovered [149]. As we and others have shown that several cancer stem cell inhibitors target high CD44 cell population and that CD44 is expressed in NEPC, it is tempting to conclude that targeted inhibition of CD44 expressing cells could also prevent the progression of t-NEPC [83,150,151,152,153]. 

**Figure 2:**
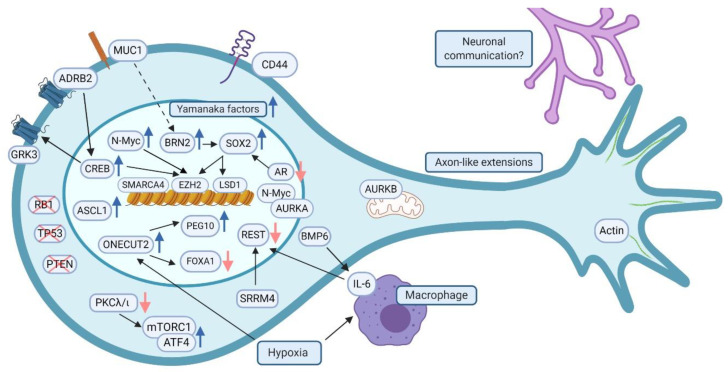
Overview of the molecular determinants of t-NEPC progression. Loss of RB1, TP53 and PTEN and increased expression of BRN2, NANOG, ASCL1, N-Myc and Yamanaka factors, such as SOX2, contribute to t-NEPC progression. CREB, activated by ADRB2, promotes EZH2 and GRK3 expression. Epigenetic modulators SMARCA4, EZH2 and LSD1 affect t-NEPC-like chromatin landscape. Increased MUC1 expression relates to increased BRN2 expression. CD44 is highly expressed in t-NEPC and it contributes to molecular interactions and signaling. BMP6 secreted by cells increases expression of IL-6 by macrophages. IL-6 and SRRM4 have inhibiting effect on REST. REST suppressed ONECUT2 expression is enhanced as a response to hypoxia/REST inhibition promoting the expression of PEG10 and SMAD3 and suppressing the expression of FOXA1. PKCλ/ι loss promotes mTORC1 complex activation and allows ATF4 to enhance the expression of genes involved in t-NEPC progression. AURKA protects N-Myc from degradation while AURKB has been shown to be involved with mitochondrial trafficking. Neuron-like appearance of t-NEPC is supported by actin, which is found in the neurite-like protrusions. Communication between neurons and cancer could be also involved in the t-NEPC progression. Figure was created with BioRender.com.

As a summary, an overview of a neuroendocrine prostate cancer cell and proposed proteins linked to t-NEPC progression and possibly regulating its neuron-like phenotype, including protein subcellular locations, is displayed in the Figure 2.

## 3. Pluripotency Transcription Factors and Neuroendocrine Plasticity

Among the transcription factors which play a role in prostate cancer neuroendocrine transdifferentiation, are the transcription factors able to produce induced pluripotent stem cells: the Yamanaka pluripotency factors OCT4, SOX2, KLF4 and MYC, along with NANOG [128]. Although induced expression of these pluripotency markers has been linked to prostate cancer treatment resistance by several research groups, surprisingly little is still known about their specific functional roles in modulating the phenotypic switch in t-NEPC. Moreover, it is unclear whether stemness is induced before or after the neuroendocrine transdifferentiation. In hippocampal neurons, SOX2 plays an important role in early steps of neurogenesis as SOX2 primes the epigenetic landscape in neural precursors enabling proper gene activation during neurogenesis [54,154,155]. Thus, similar SOX2 induced changes may be needed also in neurogenesis-like phenotype switch in t-NEPC. Interestingly, Sanchez et al., suggest that targeted therapy evasion induced stem-like phenotype in cultured LNCaP prostate cancer cells occurs after the induction of neuron-like phenotype in androgen ablated conditions whereas Nouri et al., report that cultured prostate cancer LNCaP cells display different reprogramming phenotypes depending on the cell differentiation medium used in the culture [38,39]. These findings raise questions whether the phenotypic plasticity is reversible and whether the cancer treatments induce changes that place the cells under constant phenotype-changing stage which can be modulated depending on the tumor microenvironment and stimuli-induced adaptation. It has been hypothesized that the NEPC plasticity processes could be reversed to resensitize tumors to ADT, as reported for example for EZH2 inhibitors [13]. Further studies are needed to determine whether the cellular plasticity occurs at single cell level via dynamic transdifferentiation model or if the resistant cells are enriched in heterogenous tumor in response to therapy via hierarchical model where the tumor contains a mixture of cells with different stemness or proliferative abilities [156]. Ongoing studies utilizing the single-cell RNA-sequencing and ATAC-sequencing studies will hopefully reveal more insights on the plasticity processes leading to NEPC phenotype at the single cell level. Moreover, detailed cellular imaging techniques are needed to fully understand the plasticity stages and processes at the single cell level.

Pluripotency factors have been widely studied in the context of their nuclear localization, their roles as transcription factors and how they orchestrate the chromatin remodeling and play together with the chromatin remodeling complexes. Still very little is known about their trafficking and localization in the cells and whether the control of their trafficking plays a role in cellular functions, plasticity, and phenotypic change. A recent review by van Schaijik et al., summarized the importance of the subcellular localization and potential implications on cellular functions of the stem cells markers NANOG, OCT4, SOX2, KLF4 and MYC [157]. The authors suggest that in addition to the nuclear transcriptional roles of these factors, important regulatory and function dependent roles exist depending on their subcellular localization and kinetics in the cells. For example, in comparison to the nuclear localization in the induced pluripotent stem cells, OCT4 is mostly cytoplasmic in tumor cells [157]. Additionally, SOX2 and NANOG are expressed in both the nucleus and cytoplasm in many cancer cells [157]. Thus, the functional role of the cytoplasmic expression of the pluripotency markers needs to be addressed also in relation to t-NEPC progression. Two cancer stem cell inhibitors, disulfiram and rovalpituzumab tesirine, are currently studied in clinical trials for NEPC (clinicaltrials.gov (accessed on 18 December 2020)).

## 4. The Role of Cell-Cell Communication Networks and Tumor Microenvironment in t-NEPC

### 4.1. Formation of Tunneling Microtubes

Recent evidence from different research groups and cancer models have demonstrated that intercellular communication among cancer cells and within cells in their microenvironment occur via highly dynamic membrane protrusions which form thin actin-based nanotubes between the cells. These thin membranous tubes are called either tumor microtubes, tunneling nanotubes, membrane bridges or cytonemes in the literature and they are responsible of several cargo transfer between the cells including transport of vesicles, mitochondria and other cellular components [158,159]. In prostate cancer, only one study has been described related to tunneling nanotube formation where Kretschmer et al., showed that tunneling nanotubes are observed in prostate cancer cells in response to various stress factors in vitro [158]. Kretschmer et al., reported that AR blockade as well as metabolic stresses, including acidic microenvironment (pH 6.6), serum starvation, whole nutrient starvation with HBSS/HEPES or hypoxia (1% O_2_) induce tunneling nanotubes in PC-3 and LNCaP prostate cancer cells [158]. Moreover, the LNCaP cell-formed tunneling nanotubes was also detected between unstressed prostate cancer cells or osteoblast hFOB cells suggesting that stressed prostate cancer cells are responsible of the tube formation between neighboring cells [158]. However, more studies are required to resolve whether the formation of nanotubes and neuron-like phenotype in response to stimuli have similarities, or even just describe a same phenotype seen in the cells.

### 4.2. Microenvironmental Factors, Prostate-Neuron Interactions in the Tumor Microenvironment and the Hypothesis of Prostate—Neuron Cell Fusions

Tumor microenvironment has been also shown to play a part in t-NEPC progression. Interleukin-6 (IL-6) is known to contribute to astrocytic differentiation through activation of JAK/STAT3 pathway and therefore regulating expression of genes involved in differentiation, such as glial fibrillary acidic protein (GFEP) [160]. IL-6 induced JAK/STAT3 pathway has been also shown to contribute to axon regeneration in central nervous system [160]. Co-cultures of macrophages with prostate cancer cell lines were shown to activate BMP-6/IL-6 loop, where BMP-6 secreted by prostate cancer cells induced expression of IL-6 in macrophages [161]. These co-cultured prostate cancer cell lines also had NE-characteristics. Mechanistically, IL-6 mediates its effect on neuroendocrine differentiation via suppressing RE-1 silencing transcription factor (REST) [76]. Another important microenvironmental factor in neuronal differentiation and neuroendocrine progression is TGF-β, a member of cytokine superfamily that regulates cell proliferation, apoptosis, cell differentiation [162].

Recently, it was reported that migrating progenitor cells from the central nervous system could influence the development of neuroendocrine prostate cancer [163]. Mauffrey et al., showed in Hi-MYC mouse model, that neural progenitors with doublecortin expression can travel from subventricular zone to tumor area, where they can initiate neurogenesis and generate new adrenergic neurons [163]. In xenograft models, tumors with doublecortin positive neural progenitors contributed to tumor progression dramatically and selective depletion of DCX positive cells reduced the tumor growth. However, it is not currently known whether this migration of neural progenitors from brain to tumor area also occurs in humans. Yin et al., studied more closely cancer-neural stem cell interactions and discovered that prostate cancer cells can acquire neuroendocrine features in co-cultures with neural stem cells from rat subventricular zone [164]. More specifically, the LNCaP cells used in this study lost the expression of androgen receptor and gained expression of CGA, SYP, E-cadherin and prostate specific membrane antigen (PSMA) [164]. These cells also displayed neuronal-like morphology with long axon-like extensions and it was suggested that LNCaP cells could acquire neuroendocrine features through cell-cell fusion with neural cells [164]. The authors also theorized that tumors could hijack subventricular zone to provide them with neural progenitors [164]. Further studies are needed to elaborate whether prostate cancer cell—neuron cell fusion has any consequences on t-NEPC progression and prostate cancer progression in vivo.

Schwann cells also increase tumor cell invasion in prostate and pancreatic cancer cells, and the cancer cells associate and use Schwann cells to invade and migrate along nerves in pancreatic and thyroid cancer [165,166]. Crosstalk between the tumor and central nervous system has been studied in glioma progression and tumor-induced neurogenesis, axonogenesis and immune evasion has been proposed also to play a role in prostate cancer [166,167]. However, the potential interplay between t-NEPC cells and the surrounding cells in the microenvironment has not yet been specifically studied. Moreover, in addition to proposed catecholamine synthesis induced by the immune cells in the tumor microenvironment or even neurodifferentiated prostate cells themselves, the microenvironment of prostate cancer cells may also represent a source of neuropeptide secretions including neuropeptide Y, neurotensin, vasoactive intestinal peptide, orexins and others which may also have direct impact on prostate cancer progression. For example, Zhu et al., have recently shown that neurotensin is a mediator directing tumor cell neuroendocrine differentiation. The authors discuss that neurotensin may be produced by the non-tumor cells in the tumor microenvironment and highlight that the contribution of the tumor microenvironment as a plausible source of neurotensin is still under extensive investigation [168]. Also, vasoactive intestinal peptide has been reported to induce neuroendocrine differentiation in the LNCaP prostate cancer cells and the orexin type 1 receptor is overexpressed in advanced prostate cancer with a neuroendocrine differentiation [169,170]. In contrast, low neuropeptide Y is associated with neuroendocrine development in prostate cancer [171]. These new insights into the progression of neuroendocrine prostate cancer development can also implicate new targets for treatments starting from targeting directly into proteins typically expressed in neurons or focusing on synaptic and paracrine interactions between central nervous system and cancer.

## 5. Emerging Targets and Current Clinical Trials under Investigation for NEPC

Currently, there are limited numbers of clinical trials specifically including treatment arms for NEPC or t-NEPC patients. The inhibitors currently in clinical trials include pluripotency targeting agent, cancer stem cell inhibitor disulfiram, Antabus, which we have first identified as a specific reducer of prostate cancer cell growth alone and in combination with copper or in combination with sunitinib in our high-throughput screening and preclinical studies of known drugs potentially transferable to prostate cancer targeting agents [152,172]. Another cancer stem cell inhibitor rovalpituzumab tesirine (Rova-T) is also in clinical trials (Table 1). Moreover, FOXM1 and cancer stem cell inhibitor monensin is under preclinical investigation [83,151,152]. In addition to the cancer stem cell inhibitors, clinical trials are ongoing with multiple N-Myc, AURKA and BET inhibitors and EZH2 inhibitors (clinicaltrials.gov (accessed on 18 December 2020)). Furthermore, other emerging NEPC-related inhibitors available include for example beta blockers designed to inhibit PKA-CREB1 activation via the β2-adrenergic receptor signaling, a molecular switch for neuroendocrine transdifferentiation of prostate [125]. Although beta blockers have been widely used in treatment of cardiovascular diseases, repurposing of beta blockers for prostate cancer still need mechanistic validations and specific biomarkers [125,173,174]. Other potential anti-cancer drugs that are under preclinical evaluation include an inhibitor for MUC1-C, GO-203, which blocks MUC1-C homodimerization and nuclear localization [142,143].

The list of potential drug candidates for t-NEPC is still relatively short (see Table 1), therefore additional novel targets entering clinical trials on NEPC patients are desperately needed. As an intriguing option, emerging targets from the neuronal developmental field could be translated to preclinical investigations for t-NEPC.

## 6. Future Directions

There is a substantial knowledge gap between the regulators of treatment-induced neuroendocrine prostate cancer and their relation to observed molecular mechanisms reported in the neuroscience field. In addition to finding specific novel targets and their inhibition using novel therapeutic targets, we propose following research directions under the term of ‘neurobiology of cancer’ for further investigation to fully understand and target pathways and molecules promoting both neurodevelopmental processes and neuroendocrine plasticity in prostate cancer.

### 6.1. Are Neurodevelopmental Processes Truly Activated in Neuroendocrine Differentiation?

In this review, we highlight several reported recent NEPC markers and regulators in the context of their roles in modulating neurodevelopmental processes in neurons. Many of these examples combined were derived from differential biological context and it remains unclear whether similar processes that occur in neuronal cells have similar functions in neuroendocrine prostate cancer cells. Yet, fundamental investigations are needed to understand whether the neuroendocrine plasticity leads to neurodevelopmental processes in prostate cancer treatment resistance.

### 6.2. What Is the Role of Tumor Microenvironment in Neuron-Like Phenotypic Plasticity, Do Neuroendocrine Prostate Cancer Cells Connect to Neurons and Are They Able to Establish Functional Cancer-Neuron Connections?

It is interesting that prostate cancer cells transdifferentiate to morphologically neuron-like cells in the process when they become treatment resistant. It is unknown what are the advantages in the phenotypic change especially to neuron-like cells and what are the roles of tumor microenvironment and surrounding stromal cells in controlling cellular plasticity and in the emerge of treatment-resistant aggressive forms of prostate cancer. To better understand the processes, a systematic investigation is necessary to define the roles and advantages of the neuron-like phenotype and whether the cells possessing this phenotype are truly able to connect to each other and/or to communicate to surrounding stromal neurons in the tumor microenvironment. Moreover, more investigations are required to understand whether the potential cancer cell—neuron connections are needed to support tumor growth and aggressiveness, or if there are any other advantages for the tumor cells to establish these potential connections.

### 6.3. Does the Neuroendocrine Phenotypic Plasticity in Prostate Cancer Harbor Cellular Transdiffention Processes Similar as Are Seen in the Induced Pluripotent Stem Cells and Their Transdifferentiation to Functional Neurons?

It is also interesting that the transcription factors needed for the induction of pluripotent stem cells from somatic cells in laboratory, such as Yamanaka factors, are also overexpressed or activated in neuroendocrine prostate cancer cells. As these transcription factors are able to transdifferentiate somatic cells to either pluripotent stem cells, or even straight to neurons, it is interesting to find out if these similar processes occur in prostate cancer progression—and which are the molecular mechanisms of these transdifferentiation processes in androgen deprivation induced phenotypic change and whether these processes are able to transdifferentiate the cells to functional neurons.

### 6.4. What Are the Key Players for the Phenotypic Switch? Are There Any Modulators of the Transdifferenation Process Available beyond Transcription Factors?

Transcription factors affect multiple target genes and thus are attractive therapeutic targets. However, many transcription factors are undruggable and thus targeting specific phosphatases and kinases of the signaling pathways of NEPC may be better option for drug target development. Epigenetic modulators and histone deacetylases are one option to go forward, though specific molecular mechanisms especially in t-NEPC need to be discovered. Another option is to prevent the neurodevelopmental processes like axonogenesis and neurogenesis by blocking specific downstream signaling molecules leading to the cellular phenotypic switch in neuroendocrine transdifferentiation. Moreover, advanced single cell RNA-sequencing and ATAC-sequencing analyses as well as detailed cellular imaging studies in individual cells should be addressed in addition to the current DNA and RNA based mutation and expression analyses of prostate cancer patient material which largely dominate the research field of t-NEPC development.

## 7. Conclusions

The molecular mechanisms of the treatment-induced NEPC is still poorly understood and there are no effective treatments for lethal NEPC. Here, we summarize the literature on the molecules and pathways contributing to neuroendocrine phenotype in prostate cancer in the context of their known cellular neurodevelopmental processes. Understanding of t-NEPC progression at molecular and cellular level and the comparison of the NEPC markers to known effects in neuronal processes may reveal novel mechanistic aspects of NEPC progression. Moreover, we discuss the role of tumor microenvironment and the cellular phenotypic change and propose the future directions of t-NEPC research and the opportunities to target the key regulators to inhibit the neuroendocrine plasticity. We also warrant the importance of advanced single cell RNA-sequencing and ATAC-sequencing analyses as well as cellular imaging tools in understanding the morphologic changes and the molecular phenotype switch at cellular level in t-NEPC development.

## Figures and Tables

**Table 1:** Current clinical trials under investigation for NEPC (clinicaltrials.gov (accessed on 18 December 2020)).

Agent	Target	Clinical Status
Cancer stem Cell Inhibitors		
Disulfiram	ALDH1A1 inhibitor	Phase Ib
Rovalpituzumab tesirine (Rova-T)	DLL3 targeting agent	Phase I
MYCN and AURKA inhibitors		
MLN8237	N-Myc inhibition	Phase II
Alisertib (MLN8237)	AURKA inhibition	Phase II
BET inhibitors		
ZEN003694	BET protein inhibition	Phase I
GS-5829	BET protein inhibition	Phase I and II
Epigenetic modulators/EZH2 inhibitors		
GSK2816126	EZH2 activity inhibition	Phase I
Tazemetostat (EPZ-6438)	EZH2 activity inhibition	Phase II
CPI-1205	EZH2 activity inhibition	Phase Ib and II
LSD1 inhibitor		
INCB059872	LSD1 inhibition	Phase I and II
